# Use of Medications by Breastfeeding Women in the 2015 Pelotas (Brazil) Birth Cohort Study

**DOI:** 10.3390/ijerph17020568

**Published:** 2020-01-16

**Authors:** Bárbara Heather Lutz, Diego Garcia Bassani, Vanessa Iribarrem Avena Miranda, Marysabel Pinto Telis Silveira, Sotero Serrate Mengue, Tatiane da Silva Dal Pizzol, Mariângela Freitas da Silveira, Andréa Dâmaso Bertoldi

**Affiliations:** 1 Department of Social Medicine, Faculty of Medicine, Federal University of Pelotas, Avenida Duque de Caxias, 250, Pelotas 96030-000, RS, Brazil; 2 Post-Graduate Program in Epidemiology, Federal University of Pelotas, Rua Marechal Deodoro, 1160, Pelotas 96020-220, RS, Brazil; vanessairi@gmail.com (V.I.A.M.); marysabelfarmacologia@gmail.com (M.P.T.S.); mariangelafreitassilveira@gmail.com (M.F.d.S.); andreadamaso.epi@gmail.com (A.D.B.); 3 Centre for Global Child Health, Hospital for Sick Children, 555 University Avenue, Toronto, ON M5G 1X8, Canada; diego.bassani@sickkids.ca; 4 Department of Paediatrics, University of Toronto, 555 University Avenue, Toronto, ON M5G 1X8, Canada; 5 Department of Physiology and Pharmacology, Institute of Biology, Federal University of Pelotas, Travessa André Dreyfus, s/n, Campus Capão do Leão, Pelotas 96010-900, RS, Brazil; 6 Post-Graduate Program in Epidemiology, Federal University of Porto Alegre, Av. Ipiranga, 2752, Sala 203, Porto Alegre 96020-000, RS, Brazil; sotero@ufrgs.br (S.S.M.); tatiane.silva@ufrgs.br (T.d.S.D.P.)

**Keywords:** pharmacoepidemiology, breastfeeding, drug utilization, pharmaceutical preparations, cohort studies, weaning, risk assessment

## Abstract

*Background:* This study describes medication use by women up to 3 months postpartum and evaluates the association between medication use by women who were still breastfeeding at 3 months postpartum and weaning at 6 and 12 months. *Methods:* Population-based cohort, including women who breastfed (*n* = 3988). Medications were classified according to Hale’s lactation risk categories and Brazilian Ministry of Health criteria. Duration of breastfeeding was analysed using Cox regression models and Kaplan-Meier curves, including only women who were still breastfeeding at three months postpartum. *Results:* Medication use with some risk for lactation was frequent (79.6% regarding Hale’s risk categories and 12.3% regarding Brazilian Ministry of Health criteria). We did not find statistically significant differences for weaning at 6 or 12 months between the group who did not use medication or used only compatible medications and the group who used medications with some risk for lactation, according to both criteria. *Conclusions:* Our study found no association between weaning rates across the different breastfeeding safety categories of medications in women who were still breastfeeding at three months postpartum. Therefore, women who took medications and stopped breastfeeding in the first three months postpartum because of adverse side-effects associated with medications could not be addressed in this analysis.

## 1. Introduction

The many benefits of breastfeeding for both mother and child are widely recognized. Longer duration of breastfeeding is associated with lower morbidity and mortality and higher performance in intelligence tests compared to shorter breastfeeding duration or no breastfeeding [1]. Likewise, the evidence also suggests that breastfed children experience lower rates of overweight and lower risk of developing diabetes in later stages of life [2]. Regarding the benefits for mothers, breastfeeding is associated with longer interpregnancy interval, lower the risk of developing diabetes, breast cancer, and ovarian cancer, and can also protect from postpartum depression [3,4,5].

However, the use of medications by mothers may influence the success of breastfeeding. Most studies suggest that more than 50% of women in the postpartum period (breastfeeding or not) use at least one medication [6]. These women may either discontinue breastfeeding or not adhere to the prescribed medication they need due to concerns about indirectly exposing the baby to the drug through breast milk [7]. If medications are necessary during lactation, the risks and benefits should be considered and they should be used in the cases where the benefits of treating the mother’s medical condition outweigh the potential risk to the breastfed infant [8].

Many women use medicines during lactation, and health care providers face the challenge of determining which medications are compatible with breastfeeding. Although there is a large amount of literature on the availability of active components of medications in the breast milk, the quality of information about their safety during lactation varies and can be conflicting [9,10]; therefore, many women can be advised by health practitioners to stop breastfeeding unnecessarily or avoid treatment without evidence-based information [11,12]. For instance, there are several controversies regarding the safety of hormonal contraceptive medication use, which is widely used by women at this stage, during lactation.

Considering the variety of medications commonly used during the postpartum period, relatively few known adverse events occur in infants and, in most cases, it is generally not necessary to discontinue breastfeeding due to mother’s use of medications [13]. Most lactating women consume few medications, and when they do, it is occasional. A recent study showed that women used an average of three different medicines during lactation [14]. In addition, although virtually all medicines are transferred to breast milk to some extent, the amount of the medication is generally small and is unlikely to have an adverse effect on the baby [13]. Despite this, there are also medications that cause significant toxicity in infants [15]; therefore, a case-by-case risk assessment should be done before the mother initiates breastfeeding or medication therapy [7].

Several factors can influence how medications are transferred into breast milk, such as maternal plasma concentration, maternal plasma protein binding, drug molecule size, ionization degree, lipid solubility and maternal pharmacogenomics [13]. And if the baby is exposed to a drug through breast milk, other factors determine whether there will be an effect: in addition to the drug’s toxicity, the age of the child and the dose received via breast milk which results from the combination of several factors including the substance’s half-life, time since medication was ingested, its oral bioavailability and the volume of milk ingested [13,16].

To assist health professionals in correctly prescribing and assessing the use of medications during breastfeeding, the Ministry of Health of Brazil, in partnership with the Brazilian Society of Paediatrics and the Brazilian Federation of Gynaecology and Obstetrics Associations (FEBRASGO), developed a manual containing basic information on drug use during the lactation period [17]. Other commonly used reference is the book Medications and Mothers’ Milk, last edited in 2018 [16]. More complete guidelines and information can be also obtained from LactMed [18] a free online database of the National Library of Medicine in the United States.

This study aimed to describe the use of medications by breastfeeding women of 2015 Pelotas (Brazil) Birth Cohort up to 3 months postpartum, to assess association between medication use in this period and weaning and to describe behaviours of women regarding breastfeeding and use of medicines.

## 2. Materials and Methods

Data from this study are part of the Pelotas Birth Cohort of 2015 (C2015), held in the city of Pelotas, RS, in southern Brazil. All the women who lived in the urban area of the municipality and gave birth in one of the five maternity wards of Pelotas from 1 January 2015 to 31 December 2015, were invited to participate in the study. Methodological details are published elsewhere [19].

In this work, we used data collected at birth (perinatal study, in the maternity wards), at 3 and 12 months after delivery. In the studies conducted at the 3rd and 12th months of life of the children, the women were interviewed in the households, responding to a standardized questionnaire about the period after birth. Most socioeconomic and demographic variables were collected in the perinatal study. The questionnaires applied are available on the website of the research center [20].

The background characteristics evaluated were: age (13–19, 20–29 and 30–47 years), skin color (white, black and brown/other), years of schooling (0–4, 5–8, 9–11 and 12 years or more), socioeconomic status (A—richest, B, C, D/E—poorest), parity (1, 2, 3 and 4 or more), number of prenatal consultations (less than 6 visits, 6 or more), marital status (with/without partner), time since previous birth, if any (<2 years, 2 to 5 years, >5 years), prepregnancy body mass index (BMI) (<18.5, 18.5–<25.0, 25.0–<30.0, ≥30.0 kg/m^2^), experience of breastfeeding the last child (yes/no), gestational age in weeks (24 to <34, 34 to <37, 37 to 38, 39 to 40, 41 or ≥42 weeks), type of delivery (normal or C-section) and postpartum depression (considering cut-off point of 12 on the Edinburgh scale). Age was collected in complete years and then categorized. Skin color was self-reported by mothers. Schooling was reported in completed years of study and later categorized. The economic classification was built according to the *Critério Classificação Econômica Brasil* 2015 (2015 Brazilian Economic Classification Criterion), developed by the *Associação Brasileira de Empresas de Pesquisa* (ABEP—Brazilian Association of Research Companies) [21]. For parity, we considered the total number of pregnancies that resulted in live births, including pregnancy resulted in the breastfeed child. Prepregnancy body mass index was calculated based on self-reported height and weight.

Information on breastfeeding was collected in the studies conducted at 3 months and 12 months postpartum. At 3 months, the information has been extracted from the following questions: “Has the child been breastfed?” and “Until what age was the child breastfed”? At 12 months of age, information on breastfeeding has been extracted from the questions: “Is the child still breastfed”? and “Until what age was the child breastfed”? “Still breastfeeding” was an option of answer to the second question in both moments. We considered age of weaning when the process of weaning ended, that is, when the breastfeeding completely stopped.

At 3 months, information about use of medications has been extracted from the following questions: “Thus far, have you used any medicine since the child’s birth”? If an affirmative answer, the names of the medications were questioned and, subsequently, for each drug reported the following questions were asked in order to characterize their use: “Who indicated this medicine for you”? (Doctor/dentist/other person or own self); “How many days did you use this medicine”? (Up to 7 days/8–14 days/15–30 days/31–60 days/more than 60 days). Women were also asked to show medication packaging or prescriptions in order to qualify the information.

At 12 months, the following questions were also addressed to identify women’s behaviours related to breastfeeding and drug use: “Since the child was born, did you choose not to use any medication because you were breastfeeding? What is this medicine?”; “Did you stop breastfeeding because of some medicine you were using? What is this medicine?”; “The reason the child was never breastfed was because of some medicine you needed to use? What is this medicine”?

Women with HIV (human immunodeficiency virus) diagnosis were excluded from the analysis since this is an absolute contraindication to breastfeeding in Brazil [22].

The medications were classified by the Anatomical Therapeutic Chemical classification system (ATC) [23] at levels 1 (anatomical group), 2 (therapeutic group) and 5 (chemical substance).

The medications were also classified as to their suitability for breastfeeding, according to two references, described below. The first one was Hale’s Lactation Risk Categories [16], in which a medicine can be classified in 5 ways: L1—compatible with breastfeeding; L2—probably compatible; L3—probably compatible; L4—potentially hazardous and L5—hazardous.

In the L1 category are medications that have been taken by a large number of breastfeeding mothers without any observed increase in adverse effects in the infants. Controlled studies failed to demonstrate risks to breastfed children, or the product is not orally bioavailable for them. The L2 category includes medications that have been studied in a limited number of breastfeeding women without an increase in adverse effects in the infant and/or the evidence of a demonstrated risk that is likely to follow the use of these medications in a breastfeeding woman is remote. About the medications classified in L3 category, there are no controlled studies in breastfeeding women; however, the risk of untoward effects to a breastfed infant is possible, or controlled studies show only minimal non-threatening adverse effects. Medications in this category should be used only if the potential benefits justify the potential risks to the infant. Besides that, new medications that do not have published data are in this category. The L4 category includes medications with positive evidence of risk to a breastfed infant or to breast milk production, but the benefits from use in breastfeeding mothers may be acceptable despite the risk to the infant. For the medications included in the L5 category, studies in breastfeeding mothers have demonstrated that there is a significant and documented risk to the infant, or the medications have a high risk of causing damage to the breastfed child. The risks of using these medications outweigh any possible benefit from breastfeeding.

The other reference used was the classification of the Brazilian Ministry of Health [17], which considers three categories: compatible with breastfeeding, judicious use and contraindicated use during breastfeeding. The category “compatible” includes medications whose use is potentially safe during lactation since there are no reports of significant pharmacological effects for the infant. In the category “judicious use” there are medications whose use in the lactation period depends on the risk/benefit assessment. When used, they require clinical and/or laboratory monitoring of the infant and should be used for the shortest possible time and in the lowest possible dose. New medications whose safety during breastfeeding has not yet been properly documented also are in this category. The category “contraindicated use” includes medications that require discontinuation of breastfeeding due to evidence of significant risk or significant side effects in the infant.

In both criteria, for the combinations of two or more drugs, the most dangerous classification among them was considered. However, if one of the pharmaceutical ingredients of a combination drug was not included in a criterion (Brazilian Ministry of Health or Hale lactation risk categories), we chose not to classify the medication regarding that criterion. If a woman consumed several medications classified into different categories, we considered the most harmful drug used as her classification.

We performed the sample description and calculation of the prevalence of drug use according to the independent variables, including respective 95% confidence intervals and *p*-values, using the Pearson’s chi-squared test. Proportions of the most commonly used drugs were also calculated and shown according to ATC classification level 1 and both breastfeeding suitability classifications. For those analyses, we included all women who ever breastfed their children.

Duration of breastfeeding was analysed according to the exposure variables using Cox regression models and Kaplan-Meier curves, with two cut-off points (at 6 and 12 months of age). For those analysis, women who stopped breastfeeding their children before 3 months of age were excluded to ensure temporality, since we had no exact information about when the women used the medications within the first three months postpartum. The adjusted analyses to verify the association between drug use and weaning were conducted using Cox regression in five hierarchical levels, with demographic and socioeconomic variables (skin color, age, schooling, socioeconomic status, and marital status) at the first level. At the second level, the variables parity, time since previous birth, prepregnancy BMI and previous experience of breastfeeding (last child) were included. At the third level, the variables were gestational age, type of delivery and number of prenatal consultations. In the fourth level, we included the variable postpartum depression. The variables age, years of schooling, prepregnancy body mass index, parity, gestational age and score at Edinburgh Scale (postpartum depression) were analysed in the continuous format. The classification of the medications used by the women (regarding Hal’s lactation risk categories and the Brazilian Ministry of Health classification) constituted the fifth hierarchical level.

For this analysis, the women in the sample were divided into two groups: those who did not use drugs or used only breastfeeding compatible medicines and those who used drugs of judicious use and drugs harmful to breastfeeding (as classified by the Brazilian Ministry of Health). Similarly, when analyzing Hale lactation risk categories, we grouped women who did not use medications with those who used only L1 category drugs and compared them to women who used L2 to L5 category drugs, which represent some risk for breastfeeding.

Only variables whose *p*-value was less than 0.20 were kept in the model, ensuring control of possible confounding factors for variables at the same level and at a higher level. The level of significance adopted to consider statistically significant associations was 0.05.

Additionally, an analysis was carried out to verify whether hormonal contraceptive use acts as a risk factor for weaning. The adjusted analyses were conducted using Poisson with adjust for robust variance regression in five hierarchical levels, as described above. The contraceptive use by the breastfeeding women constituted the fifth hierarchical level. Data analysis was performed using the statistical software STATA^®^, version 12.1 (StataCorp., College Station, TX, USA).

The study was approved by the Ethics Committee of Superior School of Physical Education at the Federal University of Pelotas (ethical approval file 522.064). All interviews were performed after written informed consent by the mothers.

## 3. Results

**Figure 1:**
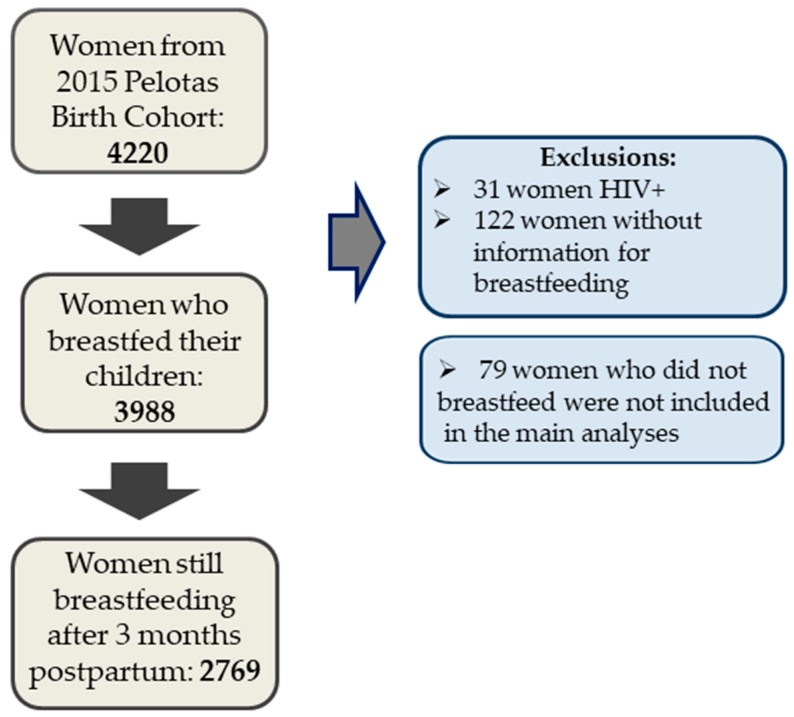
Study participant flowchart. HIV: human immunodeficiency virus.

This study presents data from 4067 mothers and their children. Women with HIV diagnosis were excluded from all analysis (N = 31), as well as women without information regarding breastfeeding (N = 122). Figure 1 shows the flowchart describing number of participants.

Table 1 shows the characteristics of the women who ever breastfed their children (*n* = 3988), according to the background variables and the prevalence of medication use in the 3 first months postpartum among women in each category. Almost half (47.6%) of the sample was between 20 and 29 years of age, a third had 9 to 11 years of formal education (34.5%), 49.7% were classified within socioeconomic level C and 85.9% had a partner. Prepregnancy BMI ranged between 18.5 and 25.0 kg/m^2^ for half (49.3%) of the sample and 86.9% attended six or more prenatal appointments. Half of the women included in this analysis were primiparae (49.8%), and 48.3% of those who had more than one child had inter-partal interval of five years or more (since the last birth). Among these women, 89.3% breastfed their previous child.

Seventy-eight percent of the pregnancies were at term (37 to 40 weeks) and 64.5% of the deliveries were C-sections; and 13.4% of the sample had a score equal to or above 12 points in the Edinburgh test, defined as a positive screening for postpartum depression.

Among the study participants who breastfed their children, 89.2% (N = 3519) reported having used any medication from birth to 3 months of age. The use of medications was more common among those women who had more years of formal education (9–11; 12 or more), who had a higher income (socioeconomic level A–B), who were living with a partner, who had a C-section and who attended 6 or more prenatal consultations. Medication use was also slightly higher among white women and among women who did not suffer from postpartum depression. Medication use was less common among younger women (≤19 years old, compared to those who were 30 years old or more, but without difference with the intermediate group of age) and among those who had 4 births or more (compared with those who had one or two children). There was no statistically significant difference in drug use regarding the other characteristics evaluated (pre-pregnancy BMI, time since previous birth, previous experience of breastfeeding and gestational age) (Table 1).

In total, 9777 medications were used by breastfeeding women, corresponding to 195 different chemical names. Table 2 shows the medications most consumed by these women, classified according to ATC level 1, Hale’s lactation risk categories [16] and the Brazilian Ministry of Health criteria [17]. The classes of medications most used, according to ATC level 1, were medications for the nervous system (N = 2448, 29.0% of medications used), medications for the genitourinary system and sex hormones (N = 1910, 22.6%) and medications for blood and hematopoietic organs (N = 1371, 16.2%). Acetaminophen was the most widely used drug (N = 1857), followed by ferrous sulphate (N = 1054) and desogestrel (N = 1016).

About the length of use, 39.3% of the medications (N = 3819) were used up to 7 days, 11.5% (N = 1119) for 8 to 14 days, 16.2% (N = 1572) for 15 to 30 days, 14.7% (N = 1426) for 31 to 60 days and 18.4% (N = 1787) for more than 60 days. Of the medication used, 91.8% (N = 8966) were prescribed by doctors or dentists and 8.2% (N = 798) were consumed by self-medication (data not shown in table).

Of the medications used, 7619 (77.9%) could be classified as to their adequacy for breastfeeding according to the criteria of the Brazilian Ministry of Health. Of these, 89.3% (N = 6805) were considered compatible with breastfeeding, 6.3% (N = 479) were considered as judicious use and 4.4% (N = 335) contraindicated for use during lactation. It was not possible to classify the remaining medications because their names were not reported (only their indication) or because the medications were not included in the criteria. In total, 331 women (10.1%) used at least one drug classified as contraindicated, 400 women (12.2%) consumed at least one drug of judicious use, and 2561 women (77.8%) only used breastfeeding-compatible medications.

Among all medications used, 7861 (80.4%) could be classified according to Hale’s lactation risk categories, with 47.4% of them (N = 3725) in category L1, 14.9% (N = 1170) in category L2, 30.4% (N = 2392) in category L3, 7.3% (N = 572) in the L4 category and only 2 medications (0.03%) in the L5 category. For purposes of analysis, we have chosen to group these two medications with those of category L4. During the first three months postpartum, 532 women (16.1%) consumed at least one L4/L5 category drug, 1696 women (51.4%) had at least one L3 category drug, 399 women (12.1%) had at least one L2 category drug and 676 women (20.5%) only used medications classified as L1. The remaining medications could not be classified for the same reasons as described above.

**Figure 2:**
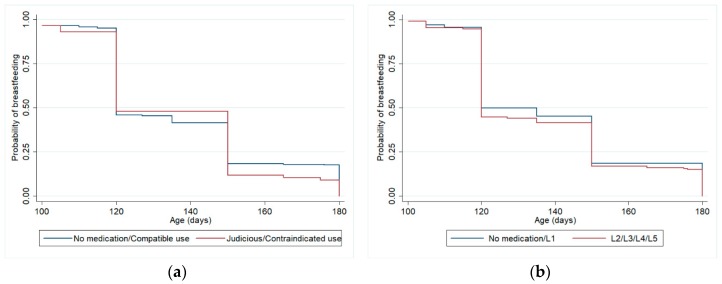
Kaplan-Meier curves (adjusted analysis *) for weaning from 3 to 6 months (excluding women who weaned before 3 months), according to maternal medication classified by Brazilian Ministry of Health Criteria (**a**) and Hale lactation risk categories (**b**). Pelotas (Brazil) Birth Cohort Study, 2015. * Analyses adjusted for maternal schooling and previous experience of breastfeeding.

**Figure 3:**
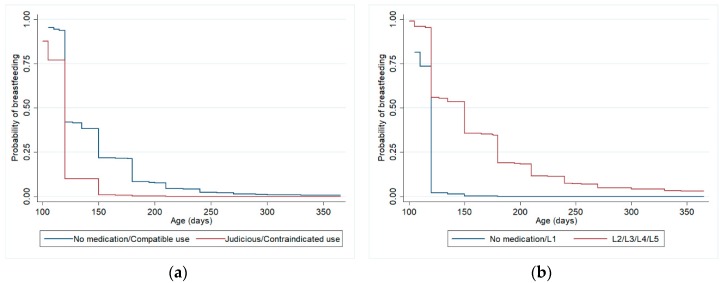
Kaplan-Meier curves (adjusted analysis *) for weaning from 3 to 12 months (excluding women who weaned before 3 months), according to maternal medication classified by Brazilian Ministry of Health Criteria (**a**) and Hale lactation risk categories (**b**). Pelotas (Brazil) Birth Cohort Study, 2015. * Analyses adjusted for maternal schooling, previous experience of breastfeeding and gestational age.

For the analysis described in Table 3, Table 4 and Table 5 and Figure 2 and Figure 3, our sample only makes up the women who were breastfeeding their children at 3 months of age (N = 2769 women), in order to ensure temporality. Among these, 21.3% (N = 589) weaned their children from 3 to 6 months and 61% (N = 1689) still were breastfeeding their children at 12 months of age.

Table 3 shows the relationship between drug use by breastfeeding women during the first three months of postpartum and weaning at 6 months, controlled for confounding variables, considering Hale’s lactation risk categories and Brazilian Ministry of Health criteria, using Cox regression models. The adjustment variables that remained in this model were maternal schooling and previous experience of breastfeeding. There were no statistically significant differences for weaning at 6 months between the group of women who did not use medication or used only compatible medication and the group who used medications with some risk for lactation. Among the adjustment variables, only maternal schooling remained associated with weaning at 6 months in the final model, considering both drug classification criteria (HR 0.97, *p*-value 0.002 in both analyses). Figure 2 shows Kaplan-Meier curves for the adjusted survival analysis of breastfeeding until 6 months.

Table 4 shows the relationship between medication use by women during the first three months of postpartum and weaning at 12 months, controlled for confounding variables, also considering both criteria. The adjustment variables that remained in the final model were maternal schooling, previous experience of breastfeeding and gestational age. There were no statistically significant differences for weaning at 12 months between the group of women who did not use medication or used only compatible medication and the group who used medications with some risk for lactation. Among the adjustment variables, maternal schooling (HR 1.02, *p*-value 0.034), previous experience of breastfeeding (HR 0.56, *p*-value < 0.01) and gestational age (HR 0.94, *p*-value 0.002) remained associated with weaning at 12 months, when analyzing drug use according to Hale’s lactation risk categories. When analyzing the use of medications according to the Brazilian Ministry of Health criteria, the same variables also remained associated with weaning at 12 months in the final model: maternal schooling (HR 1.02, *p*-value 0.039), previous experience of breastfeeding (HR 0.57, *p*-value <0.01) and gestational age (HR 0.94, *p*-value 0.003). Figure 3 shows Kaplan-Meier curves for the adjusted survival analysis of breastfeeding until 12 months.

Table 5 shows the risk of weaning at 6 and 12 months according to the use of hormonal contraceptives in the first 3 months postpartum, excluding mothers who did not breastfed or weaned before 3 months, obtained by Poisson’s regression with adjust for robust variance. The prevalence of use of progestogen-only contraceptives in this group was 42.6% (N = 1177), use of desogestrel 30.7% (N = 848), use of norethindrone 8.7% (N = 241), use of medroxyprogesterone 3.3% (N = 90) and use of combined contraceptives 3.8% (N = 104). The adjustment variables that remained in this model were marital status, previous experience of breastfeeding and gestational age for weaning at 6 months; and schooling, previous experience of breastfeeding and gestational age for weaning at 12 months. There was protection for weaning from 3 to 6 months between women who used the group of progestogen-only contraceptives in the adjusted analysis (PR = 0.78, 95%CI 0.62–0.98). However, this protection disappeared considering weaning until 12 months of age. There was no statistically significant association analyzing the progestogen-only contraceptives separately (desogestrel, norethindrone, and medroxyprogesterone) or combined contraceptives and weaning at 6 and 12 months.

During the 12-month follow-up, some questions were asked to verify women’s behaviours regarding the use of medication and breastfeeding. In this analysis, we also included mothers who did not breastfeed for reasons other than HIV diagnosis (N = 4067).

For the question, “Since the child was born, did you choose not to use any medication because you were breastfeeding”? 399 mothers (10.6%) answered affirmatively. When asked about the names of the drugs, the most cited were analgesics (including combinations with anti-inflammatory drugs, muscle relaxants, and decongestants, N = 107 women), contraceptives (N = 58), psychoactive medications (N = 55) and antibiotics. The most frequently mentioned drug was omeprazole (N = 11 women). Some women reported more than one drug.

Among the women who reported drugs that could be classified according to the Brazilian Ministry of Health criteria, most (N = 97, 47.3%) cited breastfeeding-compatible medications, 87 (42.4%) cited medications of judicious use and only 21 (10.2%) chose not to use contraindicated medications during breastfeeding. It was not possible to classify medications used by 48.6% of the women, because the name of the drug was not reported (only its indication) or because the medications were not included in the criteria.

Among the women who cited drugs that could be classified according to Hale lactation risk categories, 28 (13.8%) chose not to use breastfeeding-compatible medications (L1), 58 (28.6%) chose not to use probably compatible medications (L2), 68 (33.5%) reported L3 category medications, 48 (23.7%) reported potentially hazardous medications (L4), and only one woman (0.5%) reported choosing not to use a hazardous medicine for use during breastfeeding (category L5). It was not possible to classify medications used by 49.1% of the women, for the same reasons already reported.

The second question related to these behaviours was “Did you stop breastfeeding because of some medication you were using”? For this question, 104 women (2.8%) answered affirmatively. Report of use of antibiotics was the most frequent (27 women), followed by psychotropic (17 women), analgesics (16 women) and contraceptives (8 mothers). Some women reported more than one medication. Among the women who reported drugs that could be classified according to the Brazilian Ministry of Health criteria, 25 (56.8%) reported breastfeeding-compatible drugs, 16 (36.4%) reported medications of judicious use and only three women (6.8%), reported medications contraindicated during breastfeeding. It was not possible to classify the medications used by 57.7% of the women by these criteria.

Among the women who cited drugs that could be classified according to Hale’s lactation risk categories, 13% of them (N = 6) reported having stopped breastfeeding due to the use of medications of category L1, 41.3% (N = 19) due to the use of medications of category L2, 28.3% (N = 13) due to the use of medications of category L3 and 8 mothers (N = 17.4%) due to the use of medications of category L4. It was not possible to classify the medications used by 55.8% of the women by these criteria.

The third question was asked of mothers who reported not having breastfed, or had breastfed their children for only a few days (N = 137): “Was the reason the child was never breastfed because of some medicine you needed to use”? Fourteen mothers (10.2%) answered affirmatively. Some of the medications reported were insulin (L1 Hale’s risk category, compatible classification by the Ministry of Health), losartan (L3 Hale’s risk category, judicious use by the Ministry of Health), clonazepam (L3 Hale’s risk category, judicious use by the Ministry of Health), phenytoin (L2 Hale’s risk category, compatible classification by the Ministry of Health) and phenobarbital (L4 Hale’s risk category, judicious use according to the Ministry of Health).

## 4. Discussion

Breastfeeding practices can be affected by several historical, socioeconomic, cultural and individual factors [24]. During the sensitive period after childbirth, many women face health problems requiring the use of medications [6]. Although the most commonly used medications are considered safe during lactation and the adverse effects due to the maternal use of medications in breastfed children are rare, their use can be perceived as a barrier for breastfeeding for many reasons, especially due to misinterpretation of risk and pressures faced by the breastfeeding woman [10]. It is expected that maternal milk should be free from any form of contamination and that mothers should avoid any risks, many times putting their children’s needs before their own [25].

The perceived risk/benefit balance of using either prescribed or over-the-counter medications can impact on an individual’s decision to use them. For women who are breastfeeding, weighing risks and benefits is complicated by the consideration of its effects on both mother and child [25]. In addition, since pregnant and breastfeeding women are usually excluded from drug trials, safety information is usually limited or non-existent [10,25,26]. Thus, health practitioners are expected to balance the need to treat the mother for a medical condition and at the same time support breastfeeding, providing accurate and up-to-date advice [10,11].

In our study, the prevalence of any medication use among breastfeeding women in the period up to three months postpartum was 89%. This prevalence was very similar to a study conducted in the Netherlands [14] and slightly lower than two other studies conducted in Brazil [27,28]. The most commonly used therapeutic groups in our sample were medications for the nervous system, for the genitourinary system and sex hormones, and for blood and hematopoietic organs. In the group of medications for the nervous system, acetaminophen was the most widely used drug, since it is the first choice of analgesic and antipyretic during lactation. This medication has a similar classification in both criteria used in our study (compatible with breastfeeding). Among contraceptives, the use of desogestrel by about 29% of women is notable. This contraceptive has distinct risk classifications in the two criteria used. Among the drugs for blood and hematopoietic organs, ferrous sulphate was widely used, since it is a universal recommendation for prophylaxis of anaemia until the third month postpartum in Brazil [29].

Our results showed that 10.1% of women that breastfeed used at least one drug classified as contraindicated according to Brazilian Ministry of Health criteria, and 16.1% of them consumed at least one medication under the categories of “potentially hazardous” and “hazardous” according to Hale’s lactation risk categories. Among the main drugs used under these classifications, we highlight the use of metamizole (dipyrone), which is classified as compatible by the Ministry of Health criteria but is under L4 classification by the Hale’s lactation risk categories. Metamizole has been removed from the US and other countries’ markets because of possible side effects such as agranulocytosis and other blood dyscrasias [16] but is still widely used in Brazil as an analgesic and antipyretic.

We found that medication use was more common among those women who had more years of formal education, who had a higher income, who were living with a partner, who had delivered by a C-section and who attended 6 or more prenatal consultations. Women with more education and higher income tend to have greater access to information and health care, and it can lead to increased medication use. Likewise, women who attended more prenatal appointments also usually have postpartum consultations and could seek more medical attention for their health problems. The prevalence of C-sections in Brazil is high, but it also includes women with co-morbidities, who had higher risk pregnancies, thus it can lead these women to need more medication use also in the postpartum period.

One of the strengths of our work is the fact that is a population-based cohort study from a medium-sized city, with large sample size and follow-up rates thus encompassing a wide range of medications used by women in the postpartum period, both for acute and chronic diseases. Furthermore, the great advantage of survival analysis is that it allows the use of information from all participants until they develop the event or are censored. Thus survival analysis is the ideal technique for analysing binary responses in longitudinal studies that are characterized by different follow-up times between individuals and follow-up losses [30].

Another strength of this study is the use of two drug classifications according to their safety in lactation: a national classification system used in Brazil and a widely used international classification system. Current information about medicines safety during lactation is conflicting [9,10]. Some important differences between these two classifications were observed, specifically the hormonal contraceptives, pharmacological group widely used by women in reproductive age. According to the Brazilian Ministry of Health criteria, almost 78% of women who used medication used only compatible breastfeeding drugs, whereas in Hale’s classification only 20.5% of women used medicines in the equivalent category (L1). This fact exposes the substantial differences between both criteria. Despite this, in our findings, there was no statistically significant difference in weaning rates between women who did not consume medications or consumed only breastfeeding-compatible medications and among women who took medicines that pose some risk to milk production or risks for the child, for both criteria. Nowadays, there are few studies evaluating the duration of breastfeeding regarding the use of medicines [6,10]. A study carried out in Brazil using American Academy of Pediatrics classification for medicines’ compatibility with breastfeeding (2001) and Hale’s criteria (2004) with a sample of 246 women showed that duration of breastfeeding was longer in women who did not use any medication or who used only medicines compatible with breastfeeding [27].

Furthermore, our study did not find statistically significant differences between weaning rates at 12 months according to the contraceptive classes used by women in the first 3 months postpartum. We observed protection for weaning at 6 months in the progesterone class as a whole, but not when the contraceptives belonging to this class were analysed separately. For this analyse, we used Poisson regression with robust variance. It is a widely used method for the analysis of cross-sectional studies with binary outcomes and it can be an alternative to logistic regression since the prevalence ratio is more interpretable and easier to communicate than the odds ratio [31].

According to Brazilian criteria, estrogens are contraindicated for use during lactation due to the risk of reduced milk production, especially during the exclusive breastfeeding period. All progestogen-only contraceptives are considered to be compatible with breastfeeding by this criterion, with only an observation that norethindrone may reduce milk volume in some susceptible women [17].

The Hale’s lactation risk categories criteria advises that progestin-only birth control products are preferred for breastfeeding mothers, however, it classifies these contraceptives in the same category as combined oral contraceptive pills (L3—probably compatible) and classifies medroxyprogesterone as possibly hazardous (L4). Regarding medroxyprogesterone, although its use is common, it is also controversial. Depot medroxyprogesterone acetate (DMPA) may increase prolactin levels in breastfeeding mothers, thus increasing milk production. Despite this, clinical experience has found that some women may experience a decline in milk production or arrested early production following an injection of DMPA, particularly when it is used early postpartum, though there are no published data to support this. Nevertheless, this criterion guides that it might be advisable to recommend treatment with oral progestin-only contraceptives postpartum rather than DMPA, so that women who experience reduced milk supply could easily withdraw from the medication without significant loss of breastmilk supply. Only small traces of medroxyprogesterone are transferred to breastfed babies and no effect of concern has been proven [16].

Regarding estrogens, their early post-partum use may reduce volume of milk produced and the protein content, but it is variable, controversial, and depends on dose and the individual. Small amounts of estrogens may pass into breastmilk, but the effects in children appear minimal. This criterion advises that breastfeeding mothers should attempt to wait until lactation is established (6–8 weeks) prior to use of estrogen-containing oral contraceptives [16].

In another study conducted in Brazil, 4.5% of the women who stopped breastfeeding reported that the reason for this was the need to take medication, a higher prevalence than that found in our study (2.8%) [27]. In our sample, approximately 10% of mothers who did not breastfeed reported that the reason for this was the use of some medication, a considerably lower percentage than a study conducted in the Netherlands (about 38%) [14]. These differences in prevalence may be explained in part by the different study designs. Our population-based study included almost all births in the period of a year in a medium-sized city, while the other Brazilian study was conducted over a three-month period in a small city. The Dutch study was conducted with patients from a tertiary health care center.

Among the limitations of our study, there is the impossibility of classifying some drugs used by mothers regarding their safety in breastfeeding, because they were not included in any of the used criteria. Information about mothers’ use of medicines is only available up to the third month postpartum, and other medications used later may have influenced the duration of breastfeeding. However, studying the factors that may influence the establishment of lactation in the first three months postpartum is important as this is a key stage in the success of breastfeeding.

To ensure temporality, our survival analysis only included those mothers who still were breastfeeding at three months postpartum. Therefore, those women who took medications and stopped breastfeeding in the first three months postpartum because of adverse side-effects associated with these medications would not have been included in this analysis. This is likely to have limited our ability to find an association between medication use and cessation of breastfeeding after three months postpartum.

The possibility of recall bias should also be considered since our analysis was based on the mothers’ retrospective self-report and not on medical records. Likewise, our analysis does not take into account frequency of use or dosage.

## 5. Conclusions

Medication use in lactating women was common in this cohort and the well documented benefits of breastfeeding, coupled with limited information about the effect of these substances on breastfeeding may lead to unwarranted reduced medication use by mothers during this period. There is conflicting information available to health care professionals and patients to make an informed decision about medication use during breastfeeding. Our study found no association between weaning rates across the different breastfeeding safety categories of medications in women, considering weaning after three months postpartum. Further studies covering this limitation are needed, with designs that allow assessing weaning rates in a period closer to the use of medications by women.

## Figures and Tables

**Table 1:** Description of the study sample (excluding women who did not breastfeed their children), according to background variables and use of medicines in the first 3 months postpartum. Pelotas (Brazil) Birth Cohort Study, 2015 (N = 3988).

Characteristics	Sample		Use of Any Medicine			*p*-Value *
	N	%	N	%	95% CI	
Age (years) (N = 3987)						0.01
13–19	581	14.6	492	85.7	82.8–88.6	
20–29	1898	47.6	1679	89.3	87.9–90.7	
30–47	1508	37.8	1347	90.3	88.8–91.8	
Schooling (years) (N = 3987)						<0.001
0–4	355	8.8	274	79.9	75.6–84.1	
5–8	1026	25.7	859	84.8	82.6–87.0	
9–11	1376	34.5	1243	90.9	89.4–92.5	
12 or more	1234	31.0	1142	93.5	92.1–94.8	
Socioeconomic level (ABEP) (N = 3857)						<0.001
A (richest)	158	4.1	147	94.2	90.5–97.9	
B	1032	26.8	945	92.8	91.2–94.4	
C	1916	49.7	1689	88.8	87.4–90.3	
D-E (poorest)	751	19.5	633	85.1	82.5–87.6	
Skin Color (N = 3981)						0.003
White	2823	70.9	2518	90.1	89.0–91.2	
Black	634	15.9	537	85.5	82.7–88.3	
Mixed/other	524	13.2	457	88.4	85.6–91.2	
Marital status (N = 3987)						<0.001
Living without a partner	561	14.1	470	84.4	81.4–87.4	
Living with a partner	3426	85.9	3048	90.0	89.0–91.0	
Pre-pregnancy body mass index (kg/m^2^)^2^ (N = 3871)						0.908
<18.5	144	3.7	126	87.5	82.0–93.0	
18.5–<25.0	1907	49.3	1681	89.3	87.9–90.7	
25.0–<30.0	1083	28.0	957	89.5	87.7–91.4	
≥30.0	737	19.0	655	89.2	87.0–91.5	
Parity (only live births) (N = 3986)						0.001
1	1985	49.8	1770	90.0	88.7–91.3	
2	1247	31.3	1116	90.3	88.6–91.9	
3	440	11.0	377	86.5	83.2–89.7	
4 or more	314	7.9	255	83.3	79.1–87.5	
Time since previous birth (if any) (N = 1376)						0.262
<2 years	229	16.6	198	87.6	83.3–91.9	
2 to 5 years	482	35.0	419	88.2	85.3–91.1	
>5 years	665	48.3	597	90.7	88.5–93.0	
Previous experience of breastfeeding						0.054
(last child, if any) (N = 2001)						
No	215	10.7	178	84.4	79.4–89.3	
Yes	1786	89.3	1570	88.8	87.4–90.3	
Gestational age (N = 3988)						0.084
Early preterm (24 to <34 weeks)	121	3.0	107	88.4	82.6–94.2	
Late preterm (34 to <37 weeks)	416	10.4	354	85.9	82.6–89.3	
Early term (37 to 38 weeks)	1498	37.6	1333	90.1	88.5–91.6	
Full term (39 to 40 weeeks)	1636	41.0	1454	89.8	88.3–91.3	
Late term (41 weeks)	288	7.2	248	86.7	82.8–90.7	
Post-term (≥42 weeks)	29	0.7	23	82.1	67.0–97.3	
Type of delivery (N = 3987)						<0.001
Normal	1417	35.5	1214	86.5	84.7–88.3	
C-section	2570	64.5	2304	90.7	89.5–91.8	
Prenatal consultation (N = 3897)						<0.001
<6	510	13.1	424	84.0	80.7–87.2	
6 or more	3387	86.9	3030	90.4	89.4–91.4	
Postpartum depression (score in Edinburgh scale) (N = 3946)						0.016
<12 points	3416	86.6	3062	89.7	88.6–90.7	
≥12 points	530	13.4	455	86.2	83.2–89.1	
**Total**	3988	100	3519	89.2	88.2–90.1	

* Pearson’s chi-squared test. CI: confidence interval.

**Table 2:** Medications most commonly used during the first three months postpartum by breastfeeding women, based on The Anatomical Therapeutic Classification System [23], Hale’s Lactation Risk Categories [16] and Brazilian Ministry of Health Classification [17]. Pelotas (Brazil) Birth Cohort Study, 2015.

Therapeutic Groups	N	%	Hale’s Categories *	Brazilian Ministry of Health Classification
N—Nervous system	2448	29.0		
Acetaminophen (paracetamol)	1857	75.9	L1	Compatible
Metamizole (dipyrone)	178	7.3	L4	Compatible
Metamizole + promethazine + adiphenine	129	5.3	L4 **	Compatible **
G—Genitourinary system and sex hormones	1910	22.6		
Desogestrel	1016	53.2	L3	Compatible
Norethindrone	286	15.0	L3	Compatible
Ethinyl estradiol + levonorgestrel	273	14.3	L3	Contraindicated
B—Blood and blood forming organs	1371	16.2		
Ferrous sulfate	1054	76.9	L1	Compatible
Associations with iron, multivitamins and minerals	111	8.1	L3	Judicious use
Associations with iron and multivitamins	92	6.7	L3	Compatible
M—Musculo-skeletal system	1187	14.1		
Diclofenac	737	62.1	L2	Compatible
Ibuprofen	163	13.7	L1	Compatible
Ketoprofen	109	9.2	L2	Compatible
J—Antiinfectives for systemic use	648	7.7		
Cephalexin	378	58.3	L1	Compatible
Amoxicillin	101	15.6	L1	Compatible
Ampicillin	17	2.6	L1	Compatible
A—Alimentary tract and metabolism	499	5.9		
Simethicone	165	33.1	L3	Not classified
Scopolamine	106	21.2	L3	Judicious use
Domperidone	104	20.8	L3	Compatible
C—Cardiovascular system	204	2.4		
Methyldopa	63	30.9	L2	Compatible
Enalapril	47	23.0	L2	Compatible
Omega-3-acid ethyl esters	22	10.8	L3	Not classified
H—Systemic hormonal preparations	82	1.0		
Levothyroxine	70	85.4	L1	Compatible
Prednisone	5	6.1	L2	Compatible
Oxytocin	3	3.7	L2	Compatible
R—Respiratory system	65	0.8		
Albuterol	14	21.5	L1	Compatible
Dexclorpheniramine	10	15.4	L3	Judicious use
Sodium chloride	6	9.2	L3	Judicious use
D—Dermatologicals	28	0.3		
Benzoxiquine + menthol + benzethonium + benzocaine	12	42.9	Not classified	Not classified
Lanolin	4	14.3	Not classified	Not classified
Mupirocin ointment	2	7.1	L1	Compatible
P—Antiparasitic products, inseticides	4	0.05		
Albendazole	2	50.0	L2	Compatible
L—Antineoplastic and immunomodulating agents	3	0.04		
Doxorubicin	1	33.3	L5	Contraindicated
Rituximab	1	33.3	L3	Contraindicated
Vincristine	1	33.3	L5	Contraindicated
S—Sensory organs	1	0.01		
Dextran + hypromellose	1	100	Not classified	Not classified
**Total**	9777 ***	100		

* L1 = compatible; L2 = probably compatible; L3 = probably compatible; L4 = potentially hazardous; L5 = hazardous. ** Adiphenine not classified in both criteria. *** This table includes only the most commonly used medications. Some medications could not be classified.

**Table 3:** Relationship between drug use during the first three months postpartum and weaning at 6 months (excluding women who weaned before 3 months), controlled for confounding variables, considering Hale’s lactation risk categories [16] and Brazilian Ministry of Health criteria [17]. Pelotas (Brazil) Birth Cohort Study, 2015.

Drug Classification		Crude	Adjusted *
		Hazard Ratio (CI 95%)	Hazard Ratio (CI 95%)
Brazilian Criteria	No medication/Compatible use	1.00	1.00
	Judicious use/Contraindicated use	1.09 (0.94–1.27)	1.09 (0.88–1.35)
Hale Criteria	No medication/L1 (Compatible)	1.00	1.00
	L2/L3/L4/L5	0.94 (0.83–1.06)	1.04 (0.89–1.23)

* Controlled for maternal schooling and previous experience of breastfeeding.

**Table 4:** Relationship between drug use during the first three months postpartum and weaning at 12 months (excluding women who weaned before 3 months), controlled for confounding variables, considering Hale’s lactation risk categories [16] and Brazilian Ministry of Health criteria [17]. Pelotas (Brazil) Birth Cohort Study, 2015.

Drug Classification		Crude	Adjusted *
		Hazard Ratio (CI 95%)	Hazard Ratio (CI 95%)
Brazilian Criteria	No medication/Compatible use	1.00	1.00
	Judicious use/Contraindicated use	0.99 (0.84–1.16)	0.88 (0.69–1.12)
Hale Criteria	No medication/L1 (Compatible)	1.00	1.00
	L2/L3/L4/L5	1.00 (0.87–1.14)	0.96 (0.80–1.15)

* Controlled for maternal schooling, previous experience of breastfeeding and gestational age.

**Table 5:** Exposure to hormonal contraceptives during the first 3 months postpartum and weaning (excluding mothers who did not breastfed or weaned before 3 months), N = 2769. Pelotas (Brazil) Birth Cohort Study, 2015.

Contraceptive	Weaning 6 Months		Weaning 12 Months	
	Crude PR (95% CI)	Adjusted PR (95% CI) *	Crude PR (95% CI)	Adjusted PR (95% CI) **
Progestogen-only (all products)	0.82 (0.70–0.96)	0.78 (0.62–0.98)	1.01 (0.92–1.12)	0.95 (0.82–1.11)
Desogestrel	0.91 (0.77–1.08)	0.86 (0.66–1.12)	1.08 (0.98–1.20)	1.01 (0.85–1.19)
Norethindrone	0.75 (0.56–1.01)	0.70 (0.46–1.06)	0.93 (0.78–1.11)	0.93 (0.72–1.19)
Medroxyprogesterone	0.84 (0.53–1.32)	1.01 (0.59–1.74)	0.79 (0.58–1.08)	0.91 (0.61–1.36)
Estrogen (combined)	1.19 (0.85–1.69)	1.06 (0.68–1.64)	1.04 (0.82–1.32)	1.08 (0.80–1.47)

* Poisson regression adjusted for marital status, previous experience of breastfeeding and gestational age. ** Poisson regression adjusted for schooling, previous experience of breastfeeding and gestational age.
